# A randomised controlled trial of an advance care planning intervention for patients with incurable cancer

**DOI:** 10.1038/s41416-018-0303-7

**Published:** 2018-10-29

**Authors:** Stephanie B. Johnson, Phyllis N. Butow, Melanie L. Bell, Karen Detering, Josephine M. Clayton, William Silvester, Belinda E. Kiely, Stephen Clarke, Lisa Vaccaro, Martin R. Stockler, Phillip Beale, Natalie Fitzgerald, Martin H. N. Tattersall

**Affiliations:** 1 Centre for Medical Psychology and Evidence-based Decision-making (CeMPED), School of Psychology and Department of Medicine, University of Sydney, Sydney, NSW Australia; 2 Mel & Enid Zuckerman College of Public Health, University of Arizona, Tucson, Arizona USA; 3 Advance Care Planning Department, Austin Hospital, Melbourne, Australia; 4 HammondCare Palliative and Supportive Care Service, Greenwich Hospital and Northern Clinical School, Kolling Institute of Medical Research, University of Sydney, Sydney, NSW Australia; 5 University of Melbourne, Melbourne, Australia; 6 National Health and Medical Research Council Clinical Trials Centre, University of Sydney, Sydney, Australia; 7 Department of Medical Oncology, Northern Clinical School, Royal North Shore Hospital Sydney, Kolling Institute of Medical Research, Sydney, Australia; 8 Medical Oncology, Sydney Local Health District (SLHD), Royal Prince Alfred Hospital (RPA), Sydney, NSW Australia

**Keywords:** Palliative care, Quality of life, Cancer

## Abstract

**Background:**

We modified and evaluated an advance care planning (ACP) intervention, which had been shown to improve compliance with patient’s end of life (EoL) wishes, in a different patient population.

**Methods:**

Patients with incurable cancer, and a Family Member (FM), were randomised one-to-one to usual care or usual care plus an ACP intervention, between April 2014 and January 2017. Oncologists and participants were non-blinded. ACP was based on the Respecting Patient Choices model, with an offer to provide individualised ranges for typical, best-case and worst-case scenarios for survival time. Seven facilitators (two oncology nurses, two nurses and three allied health professionals) delivered the intervention within 2 weeks of study enrolment. The primary outcome measure, assessed by interviewing the FM 3 months after patient death, was the FM perception that the patient’s wishes were discussed, and met.

**Results:**

Six hundred and sixty-five patients from seven Australian metropolitan oncology centres were referred for consideration by their oncologists, 444 (67%) met the study inclusion criteria and were approached by a study researcher. Two hundred and eight patients (47%) and their FM entered the trial as dyads. Fifty-three (46%) dyads in the ACP group and 63 (54%) dyads in the usual-care group had complete primary outcome data (*p* = 0.16). Seventy-nine patients and 53 FMs attended an ACP discussion. Mean length of discussion was 57 min. FMs from 23 (43%) dyads allocated to ACP and 21 (33%) dyads allocated usual care reported the patient’s EoL wishes were discussed and met (difference 10%, 95% CI: −2 to 8, *p* = 0.27). There were no differences in EoL care received, patient satisfaction with care; FM satisfaction with care or with death; or FM well being. Rates of palliative care referral were high in both groups (97% vs 96%).

**Conclusions:**

A formal ACP intervention did not increase the likelihood that EoL care was consistent with patients’ preferences.

## Introduction

Professional bodies, physicians and researchers have voiced concerns about the quality of care for cancer patients nearing death.^1,2^ Decreasing aggressive interventions, respecting patient preferences for care and meeting patients’ and families’ psychosocial needs are increasingly important targets of optimal cancer care.^3,4^ Advance care planning (ACP), a process that supports adults at any age or stage of health in understanding and sharing their personal values, life goals and preferences regarding future medical care, aims to ensure that people receive care that is consistent with these.^5^ ACP is being promoted in many developed countries as a central component of End of Life (EoL) care. Although, existing research suggests that cancer patients infrequently engage in EoL discussions.^6^

Large and well-designed studies in EoL care are both challenging and uncommon.^7^ ACP for cancer patients was found to be associated with improved quality of life, reduced use of aggressive treatments at the EoL, and increased length of hospice stays in one North American study.^8^ In another American study, Mack et al.^9^ determined that patients with cancer are more likely to receive EoL care consistent with their preferences when they have had the opportunity to discuss their wishes for EoL care with a physician. Results of non-cancer studies evaluating ACP suggest that coordinated ACP interventions may result in improvements in concordance between patients’ preferences for EoL care and the EoL care received (3 of 4 studies)_._^10,11^ However, with the majority of research to date conducted in the United States (US), and of low quality,^12,13^ there is limited evidence to support the implementation of ACP interventions in different care settings and across cultures.^10^

We report the results of a cancer-specific, multi-site Australian randomised controlled trial of an ACP intervention facilitated by a health-professional external to the treatment team. We hypothesised that ACP would: (a) increase discussion and documentation of patient wishes for EoL care and increase perceived and actual compliance with patients’ EoL wishes; (b) improve the quality of death; and (c) result in less mental and physical distress in family members.

## Methods

We conducted a prospective multi-site randomised controlled trial with two parallel groups receiving usual care plus a coordinated ACP intervention or usual care alone. The patient and a nominated family member/friend (hereafter, referred to as FM) were mailed questionnaires at baseline, and 6 weeks after completion of the ACP intervention, and then at 3 month intervals until the patient’s death or study close (3 years after commencement). FM in this context included those closest to the person in knowledge, care and affection, including family, family-in-law or friends. The FM was contacted 3 months after the patient’s death to complete a final structured interview and questionnaire. Following the patient’s death, their medical record was reviewed for documentation of EoL preferences and assessment of the care received in the final 2 weeks of life.

### Participants

Eligibility criteria included: age ≥ 18 years, diagnosis of incurable cancer, expected survival time of 3–12 months (as estimated by the treating oncologist), prior systemic anticancer therapy, and ability to complete questionnaires and have an ACP conversation in English. Patients were excluded if they had previously completed formal ACP. Participating patients were asked to nominate an adult FM to participate in the trial with them; those unable to do so were excluded from the trial.

Participants were recruited from outpatient and inpatient departments of seven metropolitan oncology centres across two Australian states (2 in Victoria and 5 in New South Wales (NSW)). Patients were informed about the study by their treating oncologist. If interested in participating, a member of the research team followed up with the patient and FM to provide greater detail about the study, and obtain written consent. Consenting participants completed the baseline study questionnaire and were then randomised to the ACP intervention or usual care.

### Randomisation and blinding

Participants were randomised centrally, using an interactive voice response system at the National Health and Medical Research Council (NHMRC) Clinical Trials Centre. Randomisation was balanced for by site using a minimisation algorithm. Assessors were blinded to the allocated treatment group, noting that intervention ACD documents might appear in patients’ medical records. Oncologists and patients were not blinded.

### ACP intervention

The ACP intervention and protocol for the study has been previously published.^14^ Seven facilitators (two oncology nurses, two nurses and 3 allied health professionals) received intervention training followed by peer mentoring and shadowing in the clinical environment. Intervention training was based on the Respecting Patient Choices model with the addition of skills in EoL communication. Participants in the intervention arm were additionally offered provision of individualised ranges for typical, best-case and worst-case scenarios for survival time based on their oncologist’s estimate of expected survival time.^15^ Core components of the intervention are outlined in Table 1. The intervention was delivered in a structured meeting between the patient, their FM and the ACP facilitator, conducted within 2 weeks of study enrolment. The ACP facilitator reviewed the patient’s medical notes and met with the patient’s oncologist prior to intervention delivery to discuss medical goals of care, appropriate treatment options and the patient’s prognosis. The intervention was audio-recorded for subsequent analysis. Oncologists were asked to review and sign completed Advance Care Directives (ACD). Patients were instructed that should their goals and wishes change at any stage, they should contact their ACP nurse to arrange another meeting.

**Table 1:** Core components of the ACP intervention

I. Negotiate an agenda for the consultation
II. Assess the patient’s and/or family’s readiness to discuss future care
III. Establish the patients’ preferred substitute decision maker(s)
IV. Explore the patients understanding of their medical situation, any unmet information needs and provide information if appropriate
V. Ask the patient for permission to discuss prognosis
If they wish to hear further information progress with providing best, worst and most likely scenarios.
VI. Explore the patient’s values, goals, priorities, hopes, fears and concerns for the future
E.g. When you look at the future: what do you hope for? What worries you?
What is most important to you? What makes your life worth living?
If you were to become more unwell in the future:
What would be most important to you?
How would like to be cared for?
Is there anything else we should know about your wishes?
VII. Explore if there are any situations, treatments or health states the patient would find unacceptable
E.g. Is there anything that you worry about happening?
What is the worst medical outcome for you, that you still feel would give you quality of life?
Can you think of any circumstances where you would prefer the focus of treatment to be on comfort rather than extending life?
VIII. Summarise your understanding of the person’s most important wishes for future care
IX. Consider any other specific treatment options relevant to the person’s circumstances
Consider medical interventions such as: ICU admissions, invasive mechanical ventilation, non-invasive ventilation, IV fluids and antibiotics, chemotherapy (discuss treatment intent)
X. Consider offering to make a recommendation for future medical care, if they were to become too sick to speak for themselves, based on their values and wishes
XI. Help the patient to document their wishes

### Measures

Patients completed measures at baseline, 8 weeks (6 weeks post-intervention), then every 3 months until death or the end of the study. Nominated family or friends completed measures at baseline, 8 weeks, every 3 months until the patient’s death and at 3 months after the patient’s death. Following the patient’s death, a review of their medical record assessed documentation of EoL preferences and medical interventions received in the final 2 weeks of life. Measures, and their timings, are presented elsewhere.^14^

#### Primary outcome

The primary outcome of this trial was FM perception that the patients’ EoL wishes were discussed, and complied with. This binary outcome was based on FM response 3 months after the patient’s death to the questions: (1) “Did the patient discuss with you any particular wishes he/she had about the care they would want to receive if they were dying”, (response options were on a five point Likert scale from 0 = “Not at all” to 5 = “Very much)”; and (2) “I am satisfied that at the end of his/her life their wishes were met” (response options were on a five point Likert scale from 0 “Strongly disagree” to 5 = “Strongly agree”). This outcome was scored positive if the FM reported that EoL wishes were discussed (“Quite a bit” or “Very much”) and if the patient’s EoL wishes were met (responses of “Agree” or “Strongly agree”). All other responses were coded as negative.

#### The documentation of patient preferences for EoL care and concordance with care received at the EoL

The main secondary outcomes were concordance between documented patient preferences for EoL care and care received at the EoL, calculated for each of the following aspects of care: place of death (hospital, home, hospice/palliative care unit, other); medical interventions in the last 2 weeks of life (CPR, intensive care unit [ICU] admissions, life prolonging treatments, surgery, mechanical ventilation and ‘other’ significant interventions) and chemotherapy in the last 4 weeks of life. Care received was assessed by review of medical records (inpatient and outpatient) at the patient’s cancer treatment hospital and affiliated palliative care unit(s). For patients who died at home it was assumed that aggressive interventions were not received. Concordance was rated positive either if: (a) patients expressed a preference for a specific intervention and received that intervention or (b) patients expressed a preference not to receive a specific intervention and did not receive that intervention.

Other secondary outcomes collected from record review included the prevalence, timing and location of EoL care documents and discussions, and receipt of aggressive treatments (hospital admission, ED admission, Intravenous Antibiotics, Dialysis). An ACD was defined as any formal document that sets out a person’s wishes about future medical treatment if they lose their capacity, which had been personally signed by the patient. ‘Medical orders’ such as a Do Not Resuscitate (DNR) order, were not recorded as ACDs. We also recorded non-formal documentation—‘other EoL documentation’—defined as evidence of discussion with the patient regarding (1) the patient’s preferences for care or (2) that these could not be elicited. For example, evidence of discussion regarding patient goals of care or preferences regarding cessation of chemotherapy in the oncology clinic was recorded as ‘other EoL documentation.’ A health professional’s recommendation for care or a ‘medical order’ were not deemed to constitute a ‘patient preference’.

Data were collected using a standardised form and manualised coding protocol. Coders received three days of training and attended weekly meetings with the study coordinator to discuss and reach consensus on scoring. Disagreements between coders or unusual cases were discussed and referred to the whole research team for final consensus. Percentage of agreement (POA) was calculated for each item and overall. Cohen’s Kappa was used to measure interrater reliability^16^. POA was above 90% for all items and overall, and Cohen’s kappa agreement was > 0.7 (excellent agreement) for all variables.

#### Quality of EoL care

Quality of EoL care was measured 3 months after the patient’s death using a 27-item structured interview adapted from tools used by Detering et al^12^ and Engelberg et al,^17^ assessing FM satisfaction with the quality of a patient’s death. Response options were on 5 point Likert scales, with higher scores indicating greater satisfaction.

#### The impact of death on surviving family members

Anxiety, depression and quality of life (QOL) of FMs were assessed using the Hospital Anxiety and Depression Scale (HADS)^18^ and the SF12 ^19^ measured at baseline, every 3 months until the patient’s death, and 3 months after death. Scores on the HADS subscales range from 0 to 21; higher scores indicate worse mental health. Australian weights were used to score the SF-12; scores on mental and physical well-being subscales range from 0 to 100; higher scores indicate better QOL. The impact of death on surviving FMs was measured using the Impact-of-Events Scale (IES)^20^ at 3 months after death. Scores on the IES range from 0 to 75; higher scores indicate greater distress.

#### Patient–family and patient–healthcare provider communication about EoL care

Patients rated Patient/FM and Patient/healthcare provider communication about EoL care using items adapted from Wright et al^8^ at baseline and 6 weeks post-intervention using a 6-point Likert scale from 0 “Not all” to 5 “Very Much” discussion.

#### Patient and caregiver satisfaction with care

Patient and FM satisfaction with care was assessed at 6 weeks post-intervention using a 5 question survey utilised in a previous trial^12^ focusing on satisfaction with information provision. Scores range from 0 to 24; higher scores indicate higher satisfaction.

#### Patient and family satisfaction with the ACP intervention

Patient and FM satisfaction with the ACP intervention was assessed using a 9-item study-developed questionnaire; higher scores indicate higher satisfaction.

#### Intervention fidelity

Intervention fidelity was coded using a standardised form and coding protocol, by two reviewers. Possible scores for adherence to content, ranged from 0 to 11.

### Sample size

In a previous trial by the investigator group^12^ EoL wishes were known and respected in 86% of the intervention group compared to 30% of controls. Assuming the same baseline rate of EoL wishes known and respected in cancer patients, and believing a doubling to 60% would influence clinical practice, we estimated that two study groups that each include 56 patients who die within the 3 year follow up period would result in the study having 90% power to detect a between-group difference with 95% certainty. A conservative estimate of mortality was 75%. To allow for incomplete data on 20% of patients and a further 10% of their FM, we proposed a sample size of 210 patients with incurable cancer.

### Statistical methods

Cross-sectional categorical and continuous outcomes were tested using chi-square and *t*-tests, respectively, to compare the ACP and usual-care arms. We tested for the effect of nesting within oncologist using mixed models and including a random effect for oncologist. These effects were estimated at 0, and were therefore excluded. Concordance of patient preferences and actual care was tested with chi-square or Fisher’s exact test (in the case of sparse data). Timing of EoL care documentation and palliative care were tested using the Wilcoxon Rank Sum test. Longitudinal outcomes, including FM anxiety and depression, physical and mental well being were estimated using linear mixed models. We estimated the difference between arms using contrasts within these models. We used logistic regression to investigate factors associated with the outcomes of EoL wishes discussed and met. Longitudinal outcomes, including family member anxiety and depression, physical and mental well being were estimated using linear mixed models. We estimated the difference between arms using contrasts within these models. The post-baseline, pre-bereavement means were estimated and compared. For post-bereavement, the change from baseline to the bereavement interview were estimated. Stress, as measured by the impact-of-events scale, was tested using a *t*-test, as it was only assessed at the bereavement interview.

We used logistic regression to investigate factors associated with the outcomes of EoL wishes discussed and met, and receipt of aggressive EoL care as defined by receipt of CPR; ventilation; admission to the ICU, admission to the ED, chemotherapy or surgery, in the last 2 weeks. These factors included the following patient factors: ACP intervention, patient age, gender, state (New South Wales, Victoria), marital status, medical training (yes/no), living alone, country of birth (Australia/not Australia), religion (yes/no), health insurance (yes/no), survival time, CPR preference, advanced care directive (yes/no), substitute decision maker, advanced care planning evidence from medical record review, self-reported communication about EoL wishes (with doctor, family, other health providers; with palliative care doctor or nurse), chose to receive information on life expectancy and the patient’s satisfaction with care total. Family characteristics tested were: age, gender marital status, medical training, education, living alone, country of birth, ethnicity, religion and health insurance.

### Sensitivity analyses for primary outcome and missing data

The primary outcome was whether EoL care wishes were discussed and met. As is common in EoL research, there was a high rate of missing data. We carried out a chi-square test to compare arms, on family members who participated in the bereavement interview, and had complete data on this outcome. This analysis makes a “missing completely at random” assumption, i.e., subjects analysed are no different from subjects who dropped out. If this is not true, estimates may be biased.^2^ Therefore, we undertook sensitivity analyses to assess the robustness of results to the assumptions made about the missing data, by assuming data were missing at random, i.e., by using multiple imputation conditioning on variables associated with the outcome, missingness, or both and performed multiple imputation under a “missing not at random” assumption, imputing missing values using the same variables as above, but from the usual-care group only (control group imputation).^21^ We also compared baseline characteristics of patients and FMs who had complete data on the primary outcome with those who did not.

## Results

**Fig. 1:**
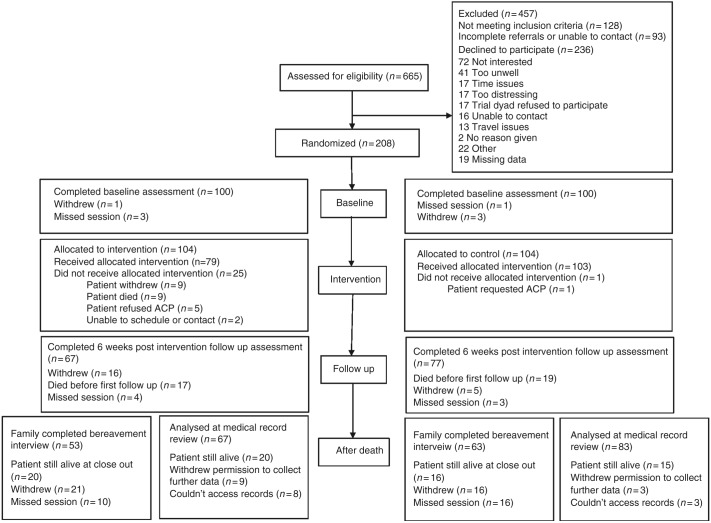
CONSORT flow diagram

Of the 665 patients referred by oncologists, 444 (67%) met the study inclusion criteria and were approached by a study researcher between April 2014 and December 2016. Two hundred and eight patients (47% of those eligible) and their nominated FMs entered the trial as dyads. (See Fig. 1: CONSORT flow diagram). Primary reasons for non-participation included ‘not being interested’ (*n* = 72) and feeling too unwell (*n* = 41). Fifty-three (46%) dyads in the ACP group and 63 (54%) dyads in the usual-care group had complete primary outcome data (*p* = 0.16). Patient data were collected between April 2014 and December 2016, and FM data between April 2014 and January 2017.

Baseline demographic and clinical variables were similar between the arms (Table 2). Patients were on average 66 years old, and FMs 58 years. Two thirds of participants were born in Australia. Genders were equally represented in patients; 73% of FMs were female. The most common cancer type was lung, followed by urological and pancreatic. Median survival of the patients was 5.4 months, with similar mortality rates in the two treatment groups (ACP 87% and usual care 84%).

**Table 2:** Demographic and clinical characteristics of participants and family members

	ACP	Usual care
Patient Characteristics		
Age Mean (range)	66 (34–90)	65 (31–82)
Months from study entry to death Median (range)	5.3 (0.4–18.3)	5.5 (0.3–22.1)
	*n* (%)	*n* (%)
Gender (male)	56 (53.9)	55 (52.9)
Married	69 (66.3)	66 (63.5)
Lives alone	20 (19.3)	11 (10.6)
Medical training (yes)	13 (12.5)	13 (12.5)
Ethnicity		
Australian-New Zealand	72 (69.2)	74 (71.2)
European	14 (13.5)	13 (12.5)
Other	14 (13.5)	13 (12.5)
Private Health Insurance (yes)	58 (55.8)	51 (49.0)
Cancer type		
Lung	30 (28.9)	28 (26.9)
Urological	13 (12.5)	6 (5.8)
Pancreas	12 (11.5)	9 (8.7)
Other GI	11 (10.6)	9 (8.7)
Gynaecological	8 (7.7)	8 (7.7)
Breast	7 (6.7)	6 (5.8)
Colorectal	6 (5.8)	17 (16.35)
Other	6 (5.8)	5 (4.8)
Missing	4 (3.9)	3 (2.9)
Skin	3 (2.9)	3 (2.9)
Head and neck	2 (1.9)	2 (1.9)
Bone and soft tissue	1 (1.0)	4 (3.9)
Unknown Primary	1 (1.0)	3 (2.9)
CNS	0 (0.0)	1 (1.0)
Family characteristics		
Age Mean (range)	58 (21–84)	58 (20–83)
	*n* (%)	*n* (%)
Male	23 (22.1)	32 (30.8)
Married	84 (80.8)	76 (73.1)
Medical training	16 (15.4)	17 (16.4)
Ethnicity		
Australian-New Zealand	78 (75.0)	77 (74.0)
European	8 (7.7)	12 (11.5)
Other	11 (10.6)	8 (7.7)
Private Health Insurance	43 (41.4)	37 (35.6)

### Advance care planning

Overall, 79 patients and 53 FMs attended an ACP discussion. Mean length of discussion was 57 min (range 18 to 120 min). Eighteen (23%) patients chose to receive individualised scenarios for expected survival time and five (6%) patients requested a second ACP meeting. Patient and FM satisfaction with ACP was high: percentage agreeing or strongly agreeing ranged from 69% to 91% for the 9 patient satisfaction items, and between 71% and 93% for the 9 FM satisfaction items. Analysis of the 55 available audiotapes produced an average score of 9.38 (SD 1.34; range 6-11) out of possible score of 11 for intervention content fidelity. Negotiating an agenda for the conversation and offering prognostic information were the intervention components most poorly adhered to (prognostic information not offered in 26% of cases). Other components were present in 74–98% of ACPs. A more detailed analysis of ACP content will be reported elsewhere.

### Primary outcome: family perception that EoL wishes were discussed and met

Of the 116 dyads with complete primary outcome data, 23 FM (43%) allocated ACP and 21 FM (33%) allocated usual care reported that the patient’s EoL wishes were discussed and met (difference 10%; 95% CI: −2 to 8, *p* = 0.27). 28 (53%) FM allocated ACP and 22 (35%) FM allocated usual care reported that EoL wishes were discussed (difference 18%, 95% CI: 0 to 36, *p* = 0.05); and 41 (79%). FM allocated ACP and 49 (78%) FM allocated usual care reported that EoL wishes were met (difference 1%, 95% CI: −14 to 16, *p* = 0.89) (Table 3). Having an ACD (OR = 3.7 (95% CI: 1.7 to 8.2, *p* = 0.001) and higher satisfaction with care (OR = 1.2 for a 1 point increase on a scale from 0 to 24, 95% CI: 1.1 to 1.4, *p* = 0.004) were significantly associated with FM report of EOL wishes being discussed and met.

**Table 3:** Primary and secondary communication outcomes

	*N*	ACP *n* (%)	Usual care *n* (%)	Difference (95% CI)	*p*-value
Family reported outcomes (bereavement interview)					
Wishes known and complied with	**116**	**23 (43)**	**21 (33)**	**10% (−2, 8)**	**0.27**
EOL wishes discussed^a^	116	28 (53)	22 (35)	18% (0, 36)	0.05
EOL wishes met	116	41 (79)	49 (78)	1 % (−14, 16)	0.89
Family satisfied with care^a^	115	46 (87)	53 (85)	−1% (−14, 11)	0.84
Family satisfied with death^a^	115	13 (25)	10 (16)	8% (−6, 23)	0.26
Patient discussed EOL care with oncologist^b^	113	38 (75)	50 (81)	−6% (−22, 9)	0.43
Family present during discussions of EOL care with oncologist^b^	67	20 (56)	19(61)	−6% (−29,18)	0.64
Family found discussions useful^b^	60	20 (65)	20 (69)	4% (−29, 19)	0.71
Family rating of the quality of discussions^c^	68	16 (52)	23 (62)	−11% (−34, 13)	0.38
Discussions with pt helpful to make decisions^b^	85	20 (49)	20 (45)	3 (−18, 25)	0.76
Patient-reported outcomes (post-intervention)					
Patient-reported communication of EOL care wishes with^b^:					
Oncologist	144	20 (30)	12 (16)	14% (6, 28)	0.04
Nominated family or friend	144	49 (73)	37 (48)	25% (10, 40)	0.002
Another health professional	144	34 (52)	16 (21)	31% (16, 46)	0.0001

^a^(Strongly agree, agree) vs (not sure, disagree, strongly disagree)

^b^(Very much, quite a bit, somewhat) vs (a little bit, not at all)

^c^(Excellent, very good, good) vs (fair, poor)

### Documentation of EoL wishes

Medical record review showed a greater prevalence of: formal ACDs (74% ACP vs 6% usual care, *p* < 0.0001); documentation of a Substitute Decision Maker (SDM) (72% vs 27%, *p* < 0.0001); and documentation of preference for Place of Death (POD) (75% vs 43%, *p* < 0.0001) in the ACP arm. Rates of discussion of preferences other than in formal ACD were high in both groups (76% vs 69%, *p* = 0.29), with no differences in EoL documentation of discussions with the patient regarding preferences for care in the clinic notes, doctors’ letters or inpatient notes, or the timing of these discussions. See Table 4.

**Table 4:** Medical record review: Concordance between documented preferences for EOL care and care received at the EoL. Difference, 95% CI, and p-values are for Documented and complied with vs (Documented not complied with or not documented)

Outcomes	*N*	ACP *n* (%)	Usual care *n* (%)	Difference %(95% CI)	*p*-value
Concordance and End of Life care received					
Actual Place of death	151				0.85
Hospital		12 (18)	16 (19)		
Home/nursing home/ hostel		13 (19)	20 (24)		
Hospice/palliative care		37 (54)	41 (49)		
Don’t know/other		6 (9)	6 (7)		
Place of death	145			24 (8, 39)	0.003
Documented and complied with		31(49)	21 (26)		
Documented not complied with		15 (24)	14 (17)		
Not documented		17 (27)	47 (57)		
CPR received in last 2 weeks	136	0	0	—	—
CPR					
Documented and complied with		42 (75)	19 (23)		
Documented not complied with		2 (4)	0 (0)		
Not documented	138	12 (21)	63 (77)	52 (37, 66)	<0.0001
ICU admission in last 2 weeks	136	1(2)	0 (0)	2 (−1, 5)	0.45
ICU admission	148			17 (4, 30)	0.008
Documented and complied with		18 (28)	9 (11)		
Documented not complied with		0 (0)	1 (1)		
Not documented		47 (72)	74 (88)		
Chemotherapy received in last 4 weeks	142	13 (21)	11 (14)	7 (−6, 20)	0.32
Chemotherapy last 4 weeks	142			2 (13, 17)	0.82
Documented and complied with		20 (31)	23 (29)		
Documented not complied with		11 (17)	9 (2)		
Not documented		33 (52)	46 (59)		
Other significant interventions in last 2 weeks	136	16 (26)	17 (23)	4 (−11, 18)	0.63
Other goals	139			−5 (−20, 10)	0.51
Documented and complied with		14 (24)	23 (29)		
Documented not complied with		6 (10)	4 (5)		
Not documented		39 (66)	53 (66)		
Surgery in last 2 weeks	151	1 (2)	1 (1)	0 (−4, 4)	1
Surgery	142			5% (2, 13)	0.14
Documented and complied with		5 (8)	2 (2)		
Documented not complied with		0 (0)	0 (0)		
Not documented		58 (92)	77 (97)		
Mechanical ventilation in last 2 weeks	143	0 (0)	0 (0)	—	—
Mechanical ventilation	144			37 (23, 52)	<0.0001
Documented and complied with		30 (49)	10 (12)		
Documented not complied with		4 (7)	0 (0)		
Not documented		27 (44)	73 (88)		
EOL care documents and palliative care					
Prevalence of formal Advance Care Directives	150	50 (74)	5 (6)	67 (56, 79)	<0.0001
Documentation of substitute decision makers at hospital	151	49 (72)	22 (27)	46 (31, 60)	<0.0001
Other EOL care documentation	151	52 (76)	57 (69)	7 (−6, 22)	0.29
Documentation of POD preference	151	50 (75)	35(43)	32 (17, 47)	<0.0001
POD preference	85				0.04
Hospital		2 (4)	3 (9)		
Home/nursing home/ hostel		16 (32)	19 (54)		
Hospice/palliative care		22 (44)	12 (34)		
Other		10 (20)	1 (3)		
Timing of Advance Care Directive (median, IQR, months before death)	55	5.0 (2.3, 8.9)	3.2 (0.8, 3.7)		0.18
Contact with palliative care	146	64 (97)	77 (96)	0 (−5, 6)	0.81
Timing of palliative care (median, IQR, months before death)	135	2.0 (.5, 6.9)	1.6 (0.9, 4.3)		0.68

Among the 85 patients with documented preferences for POD, there was a significant difference between groups in where patients wanted to die (*p* = 0.04, see Table 4). This statistical difference was likely determined by the 10 ACP vs 1 usual-care patients whose preferences could not be categorised (e.g. ‘I want to die either at home or in palliative care’).

### Medical care at the EoL

There were no differences between groups in the care received at the EoL. None or very few patients received CPR (*n* = 0), ventilation (*n* = 0) and surgery (*n* = 1). There were no differences in place of death (hospital 18% vs 19%, home 19% vs 24%, hospice/palliative care 54% vs 49%, don’t know/other 9% vs 7%, *p* = 0.85), or receipt of chemotherapy within 4 weeks of death (21% vs 14%; *p* = 0.32). There was no evidence of difference in contact with palliative care (97% vs 96%; *p* = 0.81).

### Concordance between documented preferences and medical care at the EoL

Concordance between documented preferences and EoL care received defined as yes (preferences documented and complied with) vs no (preferences documented but not complied with/preferences not documented) was higher in the ACP arm for POD (49% vs 26%, *p* = 0.003); CPR (75% vs 23%, *p* < 0.0001); ICU admission (28% vs 11%, *p* = 0.008) and ventilation (49% vs 12%, *p* < 0.0001). See Table 4. There was no difference in concordance between chemotherapy received in last 4 weeks, surgery, ‘other’ significant interventions in the last 2 weeks, or other goals of care.

### Communication with family members and HCP

More patients allocated ACP than usual care reported communication of EoL care wishes with their oncologist 6 weeks after the intervention: (20 [30%] vs 12 [16%], *p* = 0.04; nominated FM 49 [73%] vs 37 [48%], *p* = 0.002; and another health professional, 34 [52%] vs 16 [21%], *p* = 0.0001, see Table 3). There were no differences in prior communication about EoL wishes at baseline (results not shown).

### Quality of care

There was no evidence of differences between groups in: patient satisfaction with care (*p* = 0.38); FM satisfaction with care or with death; whether FM reported patient discussion of EoL care with patient’s oncologist, or whether FMs found these discussions to be of high quality or useful (see Table 3).

### After death outcomes

There was no evidence of differences between groups in FM stress, distress, physical well being before or after death. There was greater improvement in mental well being from baseline to the bereavement interview in the usual-care group, *p* = 0.006 (see Table 5).

**Table 5:** Family psychological and physical well being

Outcome	ACP mean	Usual care mean	Difference (95% CI)	*p*-value
Pre-bereavement (mean overall assessments)^a^				
Anxiety (HADS)	10.2	10.4	−0.2 (−0.6, 0.2)	0.49
Depression (HADS)	8.8	8.9	−0.1 (−0.6, 0.4)	0.42
Mental well being (SF-12)	46.1	45.6	0.5 (−3.8, 4.7)	0.83
Physical well being (SF-12	41.2	41.6	−0.4 (1.8, 1.0)	0.58
Post-bereavement (change from baseline to 3 month interview)^a^				
Anxiety (HADS)	0.9	1.1	−0.2 (−1.0, 0.6)	0.65
Depression (HADS)	−0.7	−1.2	0.5 (−0.3,10.3)	0.24
Physical well being (SF-12)	−0.8	−1.5	0.8 (−1.6, 3.1)	0.52
Mental well being (SF-12)	2.9	9.9	−7.0 (−12.0, -2.0)	0.006
Impact-of-events (IES)	23.5	23.0	0.5 (−5.4, 6.3)	0.88

^a^Possible range for anxiety and depression is 0-21; mental and physical well being is 0-100; impact of events is 0-75

Results of sensitivity analyses were similar to the primary analyses (see Supplementary file 1).

## Discussion

This ACP intervention increased prevalence of ACDs and documentation of SDM, preferred POD, and wishes regarding specific interventions such as CPR. It also increased communication between patients, oncologists and FMs as reported 6 weeks post-intervention, although FMs reported no difference at bereavement. Contrary to our hypotheses, there were no differences in FM’s perception that the patient’s wishes for EoL care were met or about the quality of death, or FM or patient satisfaction with care. There were no differences in the medical care patients received at the EoL. Differences between groups in concordance between documented preferences and EoL care received, therefore, were largely driven by differences in documentation only.^22^ There was greater improvement in mental well being from baseline to bereavement in the usual-care group, raising the possibility the intervention adversely affected FMs.

This study documents that while formal ACP did result in higher prevalence of formal ACDs and communication between patient, FM and oncologist immediately following ACP, this does not appear to impact medical care, satisfaction or well being. Similar results have been previously demonstrated in other ACP studies conducted in cancer. Jones et al.^13^ found that an ACP intervention increased discussion with FMs and with health-professionals, but intervention participants were less happy with their communication and satisfaction with healthcare was lower. Wright et al^8^ found that EoL discussions did not affect rates of major depressive disorder, or ‘worry’ in patients, and Stein et al.^23^ found no differences in patient knowledge or patient anxiety or depression between groups randomised to receive an ACP intervention. These findings are consistent with previous reviews of the ACP literature, which have concluded the evidence for the impact of ACP on satisfaction with healthcare, decisional conflict, anxiety and depression to be limited or equivocal.^10,11^ This indicates that, while patient and family well -being are often cited as important goals of ACP, ACP interventions may be of limited benefit in regards to patient or FM well being.

These results are in contrast to two previous studies in cancer that found ACP to increase the frequency of out-of-hospital^8,9,23^ and out-of-ICU care.^8^ In our study, prudent use of aggressive interventions at the EoL, documented refusals of treatment (e.g. DNR forms), and referral to palliative care services all appeared to be routine. In this Australian cancer-specific study, almost no patients (regardless of allocated arm) received aggressive care. No patients received CPR or ventilation, ~80% died outside of acute hospital and satisfaction with care was high in both groups. Almost all had contact with palliative care and documented discussions with healthcare providers several months prior to death, and FM perception that wishes were met was high (78% vs 79%). ACP interventions may, therefore, have limited impact on medical care in settings where aggressive EoL care is already rare and there is routine access to palliative care.

Three studies outside of the oncology setting have found ACP improves compliance with EoL wishes.^12,24,25^ It is not possible to reliably determine the cause of such difference due to differences in methodology and the intervention.^22^ It is possible that the study intervention failed to increase compliance with EoL wishes in a setting where satisfaction with care was high, and documented discussions of EoL wishes, routine. It is also possible that the study intervention may not have been suitable for the cancer setting. ‘Respecting patient Choices’ has been widely used and evaluated.^12,26^ However, three quarters of our intervention participants completed ACP, half documented their preferences, and 18 accepted information regarding life expectancy. The mean length of the discussions was 57 min with a range of 18–120 min. It is likely that the range in length of meeting is dependent upon the patient and/or FMs engagement in the intervention, or possibly on the particular style and skill of the ACP facilitator. Offering life expectancy information was the least reliable measure for intervention standardisation, indicating possible facilitator reluctance, or patient reluctance (patients not wishing to receive this information, or preferring to discuss this with their oncologist) and/or sub-optimal timing of the conversations (a median of 5.5 months before death). This may represent intractable problems with ACP (some patients may not wish to engage in ACP, the optimal timing of conversations is contested and interventions are difficult to standardise), and suggest further that the physician-patient relationship may play a central role in the quality of EoL care planning. ACP programmes, facilitated by health professionals external to the treating team, therefore may not be ideal. Ongoing development in the field points to the need for an ongoing process of integrated discussion, and our results support this conclusion.

There was greater improvement in mental well being in the usual-care group compared to the intervention group. It is possible that patients who have discussed their wishes become less satisfied with their overall care, if compliance with those wishes is not met. Other ACP studies in cancer have found similar results.^27^ It is also possible that this is a type I error, given the large number of comparisons undertaken.

## Limitations

This study has important limitations. There was a high proportion of missing primary outcome data, as is common in EoL studies. Although sensitivity analyses did not reveal any obvious bias, results should be interpreted with this in mind. Nearly all participants had contact with palliative care and study questionnaires may have prompted control participants to discuss EoL preferences. Oncologists participating in the study also treated patients in both groups, raising the possibility of contamination of results. Furthermore, it is likely that a referral bias exists. Oncologists may have referred patients most receptive to ACP conversations, and rates of referral to palliative care were high (available data suggests that ~80% of Australian Cancer patients are admitted to palliative care during their final hospital admission.^28^ Therefore there are inherent sources of bias.

This study also has notable methodological strengths. The study employed a multi-site RCT design, in the absence of any gold standard measures to assess concordance tested reliability of non-validated tools and paid attention to protocol adherence through audiotape analyses of the ACP discussions. Rates of drop out and missing data are acceptable for an EoL study^29^ and rigorous sensitivity analyses to test the robustness of results were conducted.

None-the-less, our results raise important questions about the content, structure and efficacy of formal ACP interventions. Further evidence is needed to establish an evidence base for the capacity of ACP to influence the quality of EoL care across cultures, and care settings. As future steps, we recommend research should be based on a clear conceptual model of ACP, which considers the role of the patient-doctor relationship. Studies should pay attention to biases, response rates, patient and family reported outcomes and should consider cluster randomised designs to minimise the effect of potential contamination and systemic shifts in care.

## Conclusion

We have shown that an ACP intervention for Australian patients with cancer increased communication and documentation of EoL wishes but did not affect their care at the EoL, patient well -being or family well -being. The results of this cancer-specific, randomised trial suggest that ACP interventions have limited benefits in cancer services where aggressive EoL care is rare, and access to palliative care is routine and widespread.

## Electronic supplementary material

Supplementary online content
