# Supplementary material for: A randomised controlled trial of an advance care planning intervention for patients with incurable cancer
Source: Br J Cancer. 2018 Oct 29;119(10):1182–90. doi: 10.1038/s41416-018-0303-7 (PMC6251033; doi:10.1038/s41416-018-0303-7)
Supplement: Supplementary file 1 — Supplementary online content [file 41416_2018_303_MOESM1_ESM.docx]

**Supplementary Online Content**

## Sensitivity analyses for primary outcome and missing data

**eTable1.** Comparison of participants with and without complete primary outcome data (EoL care wishes discussed and met) because Family after death interviews were not conducted (socio- demographic characteristics)

**eTable2**. Comparison of participant’s with and without complete primary outcome data (EoL care wishes discussed and met) because family after death interviews were not conducted (patient/family reported outcomes)

**eTable 3.** Factors associated with Family report that end of life wishes were discussed and met (primary outcome)

**eTable4.** Documented treatment preferences by medical treatment received

**eTable5.** Documented preference for place of death by actual place of death

## Table showing number of secondary outcomes reported and not reported in the manuscript

***eTable6. Patient outcomes reported and not reported***

***eTable7. FM outcomes reported and not reported***

This supplementary material has been provided by the authors to give readers additional information.

***eTable 1: Comparison of participants with and without complete primary outcome data (EOL care wishes discussed and met) because Family after death interviews were not conducted (socio- demographic characteristics)***

| *Outcome* | *N* | *Complete (%)*  *N=116* | *Incomplete (%)*  *N=92* | *p-value* |
| --- | --- | --- | --- | --- |
| *Patient* | | | | |
| ACP Intervention group | 208 | 53 (45.6) | 51 (55.4) | 0.16 |
| Male | 208 | 67 (57.8) | 44 (47.8) | 0.15 |
| State | 208 |  |  | 0.02 |
| New South Wales |  | 63 (54.3) | 35 (38.0) |  |
| Victoria |  | 53 (45.7) | 57 (62.0) |  |
| Age (Mean) | 201 | 65.8 | 65.3 | 0.77 |
| Married | 200 | 81 (71.1) | 54 (62.8) | 0.22 |
| Lives alone | 200 | 17 (14.8) | 14 (16.5) | 0.74 |
| Australian born | 200 | 78 (67.8) | 53 (62.4) | 0.42 |
| Ethnicity (AU-NZ) | 200 | 83 (72.2) | 63 (74.1) | 0.82 |
| Medical, nursing or allied health training | 198 | 15 (13.0) | 11 (13.25) | 0.97 |
| Private Health Insurance (Yes) | 197 | 63 (55.3) | 46 (55.4) | 0.98 |
| Religion (Yes) | 195 | 64 (57.7) | 55 (65.5) | 0.27 |
| Survival (months) | 156 | 7.0 | 12.2 | 0.25 |
| Cancer type | 201 |  |  | 0.19 |
| Lung |  | 31 (30.0) | 27 (31.4) |  |
| Colorectal |  | 10 (8.7) | 13 (15.1) |  |
| Pancreas |  | 12 (10.4) | 9 (10.5) |  |
| Urological |  | 13 (11.3) | 6 (7.0) |  |
| Patient reported cancer ‘spread’ | 193 | 100 (90.9) | 63 (75.9) | 0.004 |
| *Family* | | | | |
| ACP Intervention group | 208 | 53 (45.7) | 92 51 (55.4) | 0.16 |
| Male | 208 | 34 (29.3) | 21 (22.8) | 0.29 |
| Demographic information complete | 208 | 115 (99.14) | 84 (91.3) | 0.006 |
| Age (Mean) | 197 | 59.5 | 56.4 | 0.12 |
| Married | 195 | 99 (86.1) | 61 (76.3) | 0.08 |
| Lives alone | 194 | 6 (5.2) | 4 (5.1) | 0.96 |
| Australian born | 195 | 82 (71.3) | 56 (70.0) | 0.84 |
| Ethnicity (AU-NZ) | 194 | 91 (79.8) | 64 (80.0) | 0.73 |
| Medical, nursing or allied health training | 193 | 13 (11.4) | 20 (25.3) | 0.01 |
| Private Health Insurance (Yes) | 194 | 69 (60.5) | 45 (56.25) | 0.55 |
| Religion (Yes) | 189 | 57 (52.8) | 43 (53.8) | 0.85 |

***eTable 2: Comparison of Participant’s With and Without Complete Primary Outcome Data (EOL care wishes discussed and met) because Family After Death Interviews were Not Conducted (Patient/Family Reported Outcomes). Values shown are mean or n(%).***

|  | *N* | *Complete n=116* | *Incomplete*  *n=92* | *p-value* |
| --- | --- | --- | --- | --- |
| *Family reported outcomes (Baseline)* |  |  |  |  |
| Anxiety at baseline (HADS mean score) | 193 | 10.4 | 10.4 | 0.89 |
| Depressed mood at baseline (HADS mean score) | 193 | 8.5 | 8.2 | 0.19 |
| Physical Health (SF-12 T-score mean) | 176 | 40.5 | 41.5 | 0.39 |
| Mental Health (SF-12 T-score mean) | 176 | 51.8 | 49.9 | 0.40 |
| *Patient reported outcomes (Baseline)* |  |  |  |  |
| Reports communication regarding future care with palliative care doctor | 200 | 8 (7.0) | 5 (5.9) | 0.76 |
| Reports communication with family member regarding future care | 200 | 56 (48.7) | 33 (38.2) | 0.17 |
| *Patient reported outcomes (6 weeks post intervention)* |  |  |  |  |
| Satisfaction with care (Total score mean) | 141 | 18.8 | 19.2 | 0.56 |
| *Aggressive interventions at the end of life** |  |  |  |  |
| Received aggressive interventions in the last 2 weeks of life | 151 | 0.84 | 0.75 | 0.25 |

*receipt of aggressive EOL care was defined by receipt of CPR; mechanical ventilation; admission to the ICU, admission to the ED, chemotherapy, or surgery, in the last 2 weeks.

***eTable 3. Factors associated with Family Report that End of Life Wishes were Discussed and Met (Primary Outcome)***

| *Variable* | *Odds Ratio* | *(95% CI)* | *p-value* |
| --- | --- | --- | --- |
| *Patient* | | | |
| Age | 0.98 | (0.94, 1.02) | 0.23 |
| Gender | 1.56 | (0.73, 3.32) | 0.25 |
| Married | 0.82 | (0.36, 1.88) | 0.65 |
| Lives alone | 1.12 | (0.39, 3.20) | 0.83 |
| Medical, nursing or allied health training | 1.06 | (0.35, 3.21) | 0.91 |
| Country of birth (Australia/other) | 0.94 | (0.42, 2.10) | 0.89 |
| Private Health Insurance (Yes) | 0.93 | (0.44, 1.98) | 0.85 |
| Survival (months) | 1.04 | (0.97, 1.12) | 0.25 |
| Accepted information regarding life expectancy | 2.37 | (0.58, 9.72) | 0.23 |
| CPR preference | 0.89 | (0.77, 1.02) | 0.10 |
| Documented end of life discussion in medical records | 1.21 | (0.50, 2.91) | 0.68 |
| Received aggressive interventions at the EoL | 0.9 | (0.66, 1.24) | 0.52 |
| Documented Substitute Decision Maker in the medical record | 1.78 | (0.81, 3.87) | 0.15 |
| Advance Care Directive in medical record | 3.87 | (1.73, 8.65) | 0.001 |
| Patient reported satisfaction with care (total score) | 1.21 | (1.06, 1.38) | 0.005 |
| Communication with a palliative care doctor at baseline | 0.94 | (0.21, 4.15) | 0.94 |
| Communication with family or oncologist regarding future care at baseline | 1.61 | (0.76, 3.42) | 0.21 |
| Family | | | |
| Age | 1.00 | (0.97, 1.03) | 0.82 |
| Gender | 1.03 | (0.45, 2.35) | 0.94 |
| Married | 1.07 | (0.36, 3.16) | 0.90 |
| Lives alone | 1.12 | (0.39, 3.20) | 0.83 |
| Medical, nursing or allied health training | 0.66 | (0.19, 2.29) | 0.51 |
| Country of birth (Australia/other) | 0.72 | (0.31, 1.68) | 0.45 |
| Private Health Insurance (Yes) | 1.00 | (0.47, 2.15) | 1.00 |

***eTable4. Documented Treatment Preferences by Medical Treatment Received***

| *Preference* | *Receives Treatment* | *Avoids Treatment* | *Kappa (95% CI)* |
| --- | --- | --- | --- |
| CPR (n =56)  Yes  No | 0  0 | 9  47 | 0 |
| ICU (n =27)  Yes  No | 0  1 | 3  24 | 0.36 (-0.16, 0.89) |
| Chemotherapy (n =63)  Yes  No | 10  2 | 22  29 | 0.25 (0.06, 0.43) |
| Ventilation (n = 44)  Yes  No | 0  0 | 17  27 | 0 |

* kappa calculated by comparing (wants intervention, wants intervention if certain conditions met) with (does not want intervention)

< 0 as indicating no agreement and 0–0.20 as slight, 0.21–0.40 as fair, 0.41–0.60 as moderate, 0.61–0.80 as substantial, and 0.81–1 as almost perfect agreement.

***eTable5. Documented Preference for Place of Death by Actual Place of Death***

|  | *Actual place of death* | | | |  |
| --- | --- | --- | --- | --- | --- |
| *Preference* | *Hospital* | *Home/nursing home/ hostel* | *Hospice/*  *palliative care* | *Unknown/ other* | *Concordant* |
| Hospital | 2 | 1 | 4 | 0 | 2 |
| Home/nursing home/ hostel | 9 | 15 | 10 | 1 | 15 |
| Hospice/palliative care | 4 | 2 | 24 | 4 | 24 |
| Unknown/other | 13 | 15 | 40 | 7 | 7 |
| Total | 28 | 33 | 78 | 12 | 48 |

Kappa =0.13, 95%CI: 0.5, 0.21

Kappa without unknown/other = 0.33, 95%CI: 0.17, 0.49

## Table showing number of secondary outcomes reported and not reported in the manuscript

***eTable6. Patient outcomes reported and not reported***

| **Outcome** | **Measurement tool** | **Reported here** | **Reported elsewhere** |
| --- | --- | --- | --- |
| Demographics | Demographic questionnaire | ✓ |  |
| Patient understanding of survival time | Prognosis survey and the itool |  | ✓ |
| Patient/family/ healthcare provider communication about end of life care | EOL communication with family and healthcare providers questionnaire | ✓ |  |
| Quality of life | EQ-5D5L |  | ✓ |
| Preference for quantity or quality of life | Discrete choice experiment |  | ✓ |
| Patient satisfaction with care | Satisfaction with care survey | ✓ |  |
| Costs of ACP | Costs of care survey |  | ✓ |
| Satisfaction with intervention | Satisfaction with ACP intervention (intervention arm only) | ✓ |  |
| The documentation of patient preferences for EOL care and concordance with care received at the end of life | Medical record review form | ✓ |  |
| Prevalence, timing and location of EOL care documents; | Medical record review form | ✓ |  |

***eTable7. FM outcomes reported and not reported***

| **Domain** | **Measurement tool** | **Reported here** | **Reported elsewhere** |
| --- | --- | --- | --- |
| Demographics | Demographic questionnaire | ✓ |  |
| Quality of life | SF-12 | ✓ |  |
| Bereavement adjustment | HADS | ✓ |  |
| The impact of death on surviving family members | Impact of event scale | ✓ |  |
| Quality of end of life care | Quality of end of life and satisfaction with care questionnaire | ✓ |  |
